# Improved biological value of eggs due to the addition of pomegranate seed oil to laying-hen diets

**DOI:** 10.5194/aab-66-121-2023

**Published:** 2023-03-20

**Authors:** Şaziye Canan Bölükbaşı, Büşra Dumlu, Aycan Mutlu Yağanoğlu

**Affiliations:** Department of Animal Science, Faculty of Agriculture, Ataturk University 25240 Erzurum, Türkiye

## Abstract

In this study, the effects of the addition of pomegranate seed oil (PSO) at different levels (0, 0.5, 1, and 1.5 $\mathrm{mL}\;{\mathrm{kg}}^{-1}$ ) to laying-hen rations on performance values, egg quality criteria, egg shelf life, some enzyme activity, and the fatty acid composition of yolks were investigated. In the study, 96 Lohman LSL laying hens at 64 weeks of age were used. The trial consisted of four groups, each containing 24 hens. Chickens were given feed and water ad libitum during the 8-week experiment. The first group was the control group and was fed with a basal diet, while the other groups were fed with feeds with 0.5, 1.0, and 1.5 $\mathrm{mL}\;{\mathrm{kg}}^{-1}$ PSO added to the basal feed, respectively.

The lowest feed consumption and the highest egg weight were determined in the 1 $\mathrm{mL}\;{\mathrm{kg}}^{-1}$ PSO group. The highest feed conversion ratio, the lowest eggshell weight, and the shell-breaking strength were determined in the 0.5 $\mathrm{mL}\;{\mathrm{kg}}^{-1}$ PSO group. It was determined that the egg yolk malondialdehyde (MDA) value in the groups to which pomegranate seed oil was added was significantly lower than the control group on the 28th day of storage. The lowest glutation (GSH) and catalase values were found in the control group, and the highest total antioxidant capacity (TAC) was found in the 1 $\mathrm{mL}\;{\mathrm{kg}}^{-1}$ PSO group. It was determined that the addition of PSO to the diet significantly increased the rate of saturated fatty acids (SEFA), conjugated linoleic acid (CLA), and conjugated linolenic acids (CLnA) in yolk.

The results showed that the addition of 1 $\mathrm{mL}\;{\mathrm{kg}}^{-1}$ pomegranate seed oil to the feeds decreased feed consumption, increased egg weight, and positively affected the shelf life of the egg. In short, the addition of PSO had a positive effect on shelf life, and it increased punicic acid and CLA levels without reducing egg quality.

## 1 Introduction

It is possible for people to maintain their vital activities with adequate and balanced nutrition, and animal foods have an important place in meeting the energy, protein, vitamins, and minerals required for adequate and balanced nutrition. Poultry breeding is an important option to fill this gap due to its significant advantages compared to other livestock branches. Among animal foods, eggs are an excellent food, as they are rich in nutrients, provide excellent quality protein, and are a great source of vitamin D (Applegate, 2000).

Today, consumers' tendency to choose functional foods, which are more beneficial for their physical and mental health, has led scientists to work in this field (Babicz-Zielińska, 2006). Therefore, to meet the need for animal products, efforts to increase product quality and obtain functional products continue intensively as does yield.

Some biologically active compounds are highly effective in preventing or treating chronic diseases, such as cardiovascular diseases, diabetes, and cancer. It is possible to enrich the egg with these biologically active compounds by changing the diet of chickens (Kostogrys et al., 2017). One of these biologically active components used in research is conjugated linoleic acid (CLA), which has many health benefits. However, there have been studies reporting that CLA added to the diet of laying hens has some negative effects on egg quality (Ahn et al., 1999; Kim et al., 2007; Franczyk-Zarow et al., 2008) as well as beneficial effects (Bölükbaşı and Erhan, 2005). Recently, there has been interest in conjugated linolenic acid (CLnA), due to its very important physiological properties. There are scientific studies on the antidiabetic, anticarcinogenic, hypocholesterolemic, and anti-obesity properties of CLnA (Hennessy et al., 2011; Shinohara et al., 2012b; Nekooeian et al., 2014). The inclusion of pomegranate seed oil (PSO) in a rat diet has been reported to significantly reduce blood triglyceride concentration (Białek et al., 2014). In a limited number of studies on this subject, it has been reported that CLnA added to laying-hen diets increases egg production and egg quality (Kostogrys et al., 2017; Ngo Njembe et al., 2021a, b).

One of the most important sources of CLnA is pomegranate. Pomegranate (*Punica granatum* L.) is native to Iran and the Himalayas in northern India, and it has been cultivated since ancient times throughout the Mediterranean region of Asia, Africa, and Europe (Morton and Dowling, 1987). In Türkiye, which ranks third in the world in terms of pomegranate production, approximately 537 000 t of pomegranate was produced in 2018, and approximately 30 % of this was exported, while the rest was used for domestic consumption (TUİK, 2018). Approximately 48 % of the total weight of a pomegranate consists of the peel (Zarei et al., 2011). The unused part of the seed and bark contains bioactive components rich in polyphenols (the peel contains 44.0 % and the seed is 02.6 % phenolics), vitamins, and polyunsaturated fatty acids (Singh et al., 2002; Seeram et al., 2005). It was reported that the addition of 4 % of pomegranate peel, which is rich in bioactive components, to broiler diets using waste oil reduced the peroxide value in meat (Sadabadi et al., 2021, 2022).

Pomegranate seed oil has anti-oxidant, anti-inflammatory, anticarcinogenic, hyperlipidemic, and antimicrobial effects. The pomegranate seed contains 12 %–20 % oil, and most of the oil it contains consists of conjugated octadecatrienoic fatty acid (CLnA). Punicic acid (cis 9, trans 11, cis 13 acid) is one of the most important isomers of CLnA with a share of 75 %–80 %.

This study aimed to enrich the egg in terms of CLnA (punicic acid) by adding pomegranate seed oil to laying-hen rations and to examine the effects of PSO on performance, egg quality criteria, egg shelf life, some enzymes, and egg yolk fatty acid composition.

## 2 Materials and methods

### 2.1 Material

In this study, 96 Lohman LSL laying hens at 64 weeks of age were used. The trial consisted of four groups, each containing 24 hens (six subgroups). The first group was the control group and was fed with basal feed (Table 2), while the other groups were fed with feeds with 0.5, 1.0, and 1.5 $\mathrm{mL}\;{\mathrm{kg}}^{-1}$ pomegranate seed oil added to the basal feed, respectively. Chickens were given feed and water ad libitum during the 8-week experiment.

The feeds used in the experiment were obtained from a commercial feed company. The organic pomegranate seed oil (containing 76 % punicic acid) used in the experiment was purchased from a commercial company (Table 1).

**Table 1:** Fatty acid composition of pomegranate seed oil (%).

Fatty acids	%
16 : 0	2.05
18 : 0	1.37
18 : 1	4.10
18 : 2	4.34
18 : 3 (9c11t13t)	5.90
18 : 3 (9t11t13t)	1.01
18 : 3 (9t11t13c)	3.00
18 : 3 (9c11t13c)	75.91
20 : 0	0.51
20 : 1	0.76

**Table 2:** Composition of basal diet.

Ingredients (ground)	Composition ( $g\;{\mathrm{kg}}^{-1}$ )
Corn	520.00
Soybean meal	240.00
Wheat	118.63
Limestone	90.80
Vegetable oil	10.30
Dicalcium phosphate	11.54
Vitamin and mineral mixture ${}^{*}$	2.00
NaCL	5.00
DL-Methionine (99 % purity)	1.23
L-lysine	0.50
Total	1000.00
Chemical analysis ( $g\;{\mathrm{kg}}^{-1}$ )	
Crude protein	167.00
Calcium	37.80
Total phosphorous	6.75
Calculated analysis ( $g\;{\mathrm{kg}}^{-1}$ )	
Metabolisable energy ( $\mathrm{kcal}\;{\mathrm{kg}}^{-1}$ )	2752
Lysine	9.00
Methionine and cystine	6.20

${}^{*}$ Supplied per kilogram of diet: 12 000 IU vitamin A, 2500 IU vitamin D3, 30 IU vitamin E, 4 mg vitamin K3, 3 mg vitamin B1, 6 mg vitamin B2, 30 mg niacin, 10 mg calcium D-pantothenate, 5 mg vitamin B6, 0.015 mg vitamin B12, 1 mg folic acid, 0.050 mg D-biotin, 50 mg vitamin C, 300 mg choline chloride, 80 mg manganese, 60 mg iron, 60 mg zinc, 5 mg copper, 0.5 mg cobalt, 2 mg iodine, and 0.15 mg selenium.

### 2.2 Determination of general performance values

Feed consumption and egg production, egg weight, and damaged egg rate were determined by measurements made every 2 weeks. In addition, egg quality parameters such as shape index, shell thickness, breaking strength, white ratio, shell ratio, yolk ratio, and Haugh unit (Silversides, 1994) were determined in eggs collected from each subgroup ( n=6 ).

### 2.3 Determination of egg yolk lipid oxidation amount

At the end of the experiment, eggs from each subgroup ( n=6 ) were stored at + 4 ${}^{\circ }C$ for thiobarbituric acid reactive substance (TBARS) analysis. The oxidation levels of the samples were measured on the 1st and 28th days of storage, according to the method determined by Tarladgis et al. (1960). After the egg yolk samples were placed in a Kjeldahl flask, distilled water and 6 N HCl were added and heated by steam distillation. Then, 5 mL aliquots were withdrawn and transferred to test tubes to which 5 mL of 2-TBA solution (0.02 $\mathrm{mol}\;L^{-1}$ ) were further added. These mixtures were kept in a water bath for 35 min. The mixtures, which were cooled under tap water for 5 min after being taken from the water bath, were read in the spectrophotometer at a wavelength of 538 nm. The TBARS values were described as milligrams malonaldehyde per kilogram of yolk.

### 2.4 Enzyme activities

After taking blood samples from each group ( n=6 ) and centrifuging, the serum part was separated and kept at - 20 ${}^{\circ }C$ until analysis. In the serum samples obtained, the activity measurement of glutathione peroxidase (glutathione assay kit, no. 703002, Cayman, USA, according to Matkovics et al., 1988), malondialdehyde (MDA) (glutathione assay kit, no. 10009055, Cayman, USA; according to Yoshioka et al., 1979), catalase (Goth, 1991), superoxide dismutase enzymes (SOD assay kit, no. 706002, Cayman, USA; according to Sun et al., 1988), total antioxidant capacity (Erel, 2004), and total oxidant capacity (Erel, 2005) were analyzed with commercial kits.

### 2.5 Lipid extraction and fatty acid analysis

Eggs from each subgroup ( n=6 ) were stored at + 4 ${}^{\circ }C$ for fatty acid composition. Egg yolk of 1 g was put into a 50 mL plastic tube. Isopropanol of 22.5 mL was added and shaken vigorously. Then, 12.5 mL of hexane was added to the tube, and the mixture was stirred for an additional 3 min. The mixture was centrifuged at 4000 rpm for 5 min: the upper phase was transferred to a flask, and the lower phase was washed twice with 12.5 mL of hexane solvent. Finally, the combined organic phases were dried with sodium sulfate ( ${\mathrm{Na}}_{2}{\mathrm{SO}}_{4}$ ), and the solvent was removed in the evaporator at 30 ${}^{\circ }C$ (Tsiplakou et al., 2006).

The methylation process of the samples was performed according to Kelly et al. (1998) and was carried out within the framework of the protocol. Fatty acid methyl ester was prepared by transmethylation of egg yolk oil. Methyl acetate of 100 µL was added to a solution of 100 mg of oil in 1 mL of hexane and mixed. Then, 100 µL of methylation reactant (1.75 mL of methanol and 0.4 mL of sodium methylate aqueous solution of 5.4 M) was added to the mixture. The reaction mixture was stirred at room temperature for 10 min, and then 120 µL of hydrolysis solution (prepared by dissolving 1 g of oxalic acid in 30 mL of diethyl ether) was added to the reaction mixture to destroy excess sodium methylate. The mixture was centrifuged at 5 ${}^{\circ }C$ for 5 min, and the liquid was separated for chromatographic determination and used directly.

### 2.6 Determination of fatty acid composition

Chromatographic analyses were performed using a flame ionization detector (FID) on a Shimadzu gas chromatograph (GC) Autosystem. TR-CN100 Capillary Column ( 100m×0.25 mm ID, 0.20 µm film) was used for analysis. The working principles of the GC-FID device were as follows: detector temperature of 250 ${}^{\circ }C$ , injector temperature of 240 ${}^{\circ }C$ , split model injection, split ratio of 1/10 , helium carrier gas flow of 30.0 $\mathrm{mL}\;\min^{-1}$ (constant flow model), hydrogen gas flow of 40 $\mathrm{mL}\;\min^{-1}$ , air flow of 400 $\mathrm{mL}\;\min^{-1}$ , and injection volume of 1.0 µL

### 2.7 Determination of diet chemical composition

Crude protein, calcium, and phosphorus contents of the diet were determined in accordance with AOAC (2002)

## Statistical analysis

The differences between the groups were determined by analysis of variance (ANOVA) using the SPSS package program (SPSS 20.0). Duncan multiple comparison tests were used to compare the treatment averages.

The statistical model is as follows:

1 Yij=μ+ai+eij,

where Yij is the dependent variable, μ is the overall mean, ai is the effect of PSO, and eij is the random error.

## 3 Results and discussion

The effect of adding pomegranate seed oil to the rations on performance values is presented in Table 3. It was determined that the performance values were significantly affected by the addition of PSO. It was found that the addition of 1 $\mathrm{mL}\;{\mathrm{kg}}^{-1}$ PSO to the ration significantly reduced feed consumption but increased egg weight. The lowest egg production and feed conversion rate were observed in the 0.5 $\mathrm{mL}\;{\mathrm{kg}}^{-1}$ PSO group.

**Table 3:** Effect of pomagranate seed oil on performance in laying hens.

Groups	Feed intake (g)	Egg production (%)	Egg weight (g)	Feed conversion ratio (g : g)
Control	131.84 ${}^{a}$	84.92 ${}^{a}$	67.72 ${}^{b}$	2.32 ${}^{b}$
0.5 $\mathrm{mL}\;{\mathrm{kg}}^{-1}$ PSO	132.74 ${}^{a}$	76.80 ${}^{b}$	69.14 ${}^{\text{ab}}$	2.53 ${}^{a}$
1 $\mathrm{mL}\;{\mathrm{kg}}^{-1}$ PSO	125.59 ${}^{b}$	78.05 ${}^{\text{ab}}$	70.95 ${}^{a}$	2.30 ${}^{b}$
1.5 $\mathrm{mL}\;{\mathrm{kg}}^{-1}$ PSO	129.51 ${}^{\text{ab}}$	85.16 ${}^{a}$	67.51 ${}^{b}$	2.29 ${}^{b}$
SEM	0.950	1.360	0.460	0.030
P value	0.030	0.040	0.021	0.039

${}^{\text{a, b}}$ The column average is significantly different ( p<0.05 ). SEM: standard error of the means.

In a study by Ngo Njembe et al. (2021a), it was determined that the addition of olive oil and flaxseed and pomegranate seed oil to the ration had no effect on egg production and egg weight. However, in another study by the same researchers, it was determined that the addition of 5 % PSO to the ration decreased feed consumption compared to the olive oil-added group but increased egg production significantly (Ngo Njembe et al., 2021b).

In their study, Kostogrys et al. (2017) added different levels of punicic acid to laying-hen rations, and they found that the feed consumption, egg production, and feed conversion ratio increased with an increase in punicic acid level. In a study conducted on laying hens, the effect of adding 5 %, 10 %, and 15 % pomegranate seed pulp to the ration on performance values was investigated. As a result of the research, it was determined that 5 % pomegranate pulp increased egg production and had no effect on feed consumption, feed efficiency, and egg weight. The researchers concluded that the increase in egg production was due to the presence of punicic acid in the pomegranate seed pulp (Saki et al., 2014). The number of studies examining the effect of pomegranate seed oil on laying hens is limited. Studies conducted to date have shown that PSO has a positive effect on egg production. In this study, it was determined that the addition of 0.5 $\mathrm{mL}\;{\mathrm{kg}}^{-1}$ PSO significantly reduced egg production compared to the control group. This may be due to the fact that the level of punicic acid in the diet of the chickens in this group is lower than in the other groups, so the chickens in this group adapt to the diet later and have less bio-availability from the feed.

It was observed that the addition of PSO to the diet had no effect on shape index, shell thickness, albumen ratio, shell ratio, egg yolk, and Haugh unit (Table 4). It was found that the rate of damaged eggs and shell-breaking strength were affected by PSO. It was determined that the addition of 0.5 $\mathrm{mL}\;{\mathrm{kg}}^{-1}$ PSO to the diet significantly decreased the rate of damaged eggs and the shell-breaking strength ( P<0.05 ). In addition to studies reporting that dietary CLA levels improve egg quality (Bölükbaşı and Erhan, 2005), there are also studies that report the negative effects on egg quality such as the Hough unit, shell-breaking strength, and egg hardness (Kim et al., 2007; Franczyk-Zarow et al., 2008). In this study, the damaged egg ratio and shell-breaking strength were adversely affected only in the 0.5 $\mathrm{mL}\;{\mathrm{kg}}^{-1}$ PSO group. This may be due to the fact that groups with a higher percentage of PSO have high CLnA values as well as CLA, as there are studies reporting that CLnA improves egg quality (Kostogrys et al., 2017). Kim et al. (2007) reported that the use of CLA in combination with several other fatty acids improved egg quality. Saki et al. (2014) reported that adding different levels of pomegranate seed pulp to the ration had no effect on egg quality criteria.

**Table 4:** Effect of pomagranate seed oil on egg quality in laying hens.

Groups	Shape	Shell	Shell	Albumen	Egg yolk	Damaged	Haugh	Shell-breaking
	index	thickness	ratio	(%)	(%)	egg ratio	Unit	strength
		( µm )	(%)			(%)		( $\mathrm{kg}\;{\mathrm{cm}}^{2}$ )
Control	71.94	0.432	11.85	55.49	32.64	0.19 ${}^{b}$	86.11	2.50 ${}^{a}$
0.5 $\mathrm{mL}\;{\mathrm{kg}}^{-1}$ PSO	72.03	0.473	11.74	55.72	32.54	0.96 ${}^{a}$	89.81	2.07 ${}^{b}$
1 $\mathrm{mL}\;{\mathrm{kg}}^{-1}$ PSO	72.23	0.458	11.87	55.58	32.54	0.27 ${}^{b}$	88.60	2.44 ${}^{a}$
1.5 $\mathrm{mL}\;{\mathrm{kg}}^{-1}$ PSO	72.76	0.442	11.67	55.73	32.60	0.24 ${}^{b}$	88.62	2.57 ${}^{a}$
SEM	0.37	0.008	0.053	0.250	0.150	0.110	0.870	0.060
P value	0.884	0.299	0.218	0.149	0.193	0.021	0.468	0.016

${}^{\text{a, b}}$ The column average is significantly different ( p<0.05 ). SEM: standard error of the means.

There was no significant difference between the groups in terms of TBARS values on the 1st day of storage. However, at the end of the 4-week storage period (28th day), it was determined that the TBARS value was significantly lower ( P<0.01 ) in the groups that had added PSO to the diet compared to the control group (Table 5). The decrease in the TBARS value was found to be associated with the increase in the egg yolk of CLA and CLnA which have antioxidant effects. There are studies showing that the TBARS value in egg yolk and chicken meat decreases depending on the increase in the CLA level in the diet (Bölükbaşı and Erhan, 2005, 2007; Bölükbaşı, 2006).

**Table 5:** Effect of PSO on TBARS of egg values of yolk in laying hens ( $\mathrm{mg}\;\mathrm{MDA}\;{\mathrm{kg}}^{-1}$ ).

Groups	1 d	28 d
Control	0.430	0.483 ${}^{a}$
0.5 $\mathrm{mL}\;{\mathrm{kg}}^{-1}$ PSO	0.434	0.440 ${}^{b}$
1 $\mathrm{mL}\;{\mathrm{kg}}^{-1}$ PSO	0.428	0.446 ${}^{b}$
1.5 $\mathrm{mL}\;{\mathrm{kg}}^{-1}$ PSO	0.434	0.451 ${}^{b}$
SEM	0.004	0.005
P value	0.943	0.006

${}^{\text{a, b}}$ The column average is significantly different. SEM: standard error of the means.

Rojo-Gutiérrez et al. (2021) determined that pomegranate seed oil showed 91.29 % scavenging activity against DPPH radicals. In this study, it was determined that punicic acid in pomegranate seed oil accumulated in egg yolk at a significant level. Therefore, the TBARS values of egg yolks were found to be lower on the 28th day compared to the control group.

It has been shown in many studies that the antioxidant activity of pomegranate peels, seeds, and juice is quite high (Gil et al., 2000; Rojo-Gutiérrez et al., 2021; Singh et al., 2002; Li et al., 2006; Shabtay et al., 2008). Devatkal and Naveena (2010) observed that the TBARS value decreased significantly in their study in which they examined the effect on the shelf life of goat meat by adding 2 % pomegranate seed powder. In addition, there are many studies showing that processing chicken and red meat with pomegranate juice increases the shelf life (Naveena et al., 2008; Vaithiyanathan et al., 2011; Devatkal and Naveena, 2010).

The addition of pomegranate seed oil to the laying-hen diet was found to significantly alter serum enzymes (Table 6). It was determined that glutation (GSH) and catalase values were significantly higher ( P<0.01 ) in the PSO groups compared to the control group. The highest GSH was found in the 1.5 $\mathrm{mL}\;{\mathrm{kg}}^{-1}$ PSO group, and the highest catalase values were detected in the 0.5 and 1 $\mathrm{mL}\;{\mathrm{kg}}^{-1}$ PSO groups. It was found that the MDA value was significantly higher ( P<0.01 ) in the 1 and 1.5 $\mathrm{mL}\;{\mathrm{kg}}^{-1}$ PSO groups. It was observed that the addition of 1.5 $\mathrm{mL}\;{\mathrm{kg}}^{-1}$ PSO to the ration significantly reduced the serum SOD value ( P<0.01 ). The highest TAS (total antioxidant) value was found in the 1 $\mathrm{mL}\;{\mathrm{kg}}^{-1}$ PSO group, and the lowest TOS (total oxidant) value was found in the 0.5 $\mathrm{mL}\;{\mathrm{kg}}^{-1}$ PSO group.

**Table 6:** Effects of PSO on some antioxidant enzymes in the serum of the laying hens.

Groups	GSH	Catalase	LPO	SOD EU	TAS trolox equiv.	TOS
	$\mathrm{nmol}\;{\mathrm{mL}}^{-1}$	$µ\mathrm{mol}\;\min^{-1}\;{\mathrm{mL}}^{-1}$	$\mathrm{nmol}\;\mathrm{MDA}\;{\mathrm{mL}}^{-1}$	mL	L	
Control	1.48 ${}^{c}$	65.98 ${}^{c}$	362.22 ${}^{b}$	12.90 ${}^{a}$	0.24 ${}^{b}$	51.88 ${}^{a}$
0.5 $\mathrm{mL}\;{\mathrm{kg}}^{-1}$ PSO	1.41 ${}^{b}$	152.28 ${}^{a}$	354.44 ${}^{b}$	11.16 ${}^{a}$	0.23 ${}^{b}$	36.83 ${}^{b}$
1 $\mathrm{mL}\;{\mathrm{kg}}^{-1}$ PSO	1.54 ${}^{\text{ab}}$	159.05 ${}^{a}$	378.88 ${}^{a}$	12.82 ${}^{a}$	0.47 ${}^{a}$	42.83 ${}^{\text{ab}}$
1.5 $\mathrm{mL}\;{\mathrm{kg}}^{-1}$ PSO	1.56 ${}^{a}$	120.98 ${}^{b}$	381.11 ${}^{a}$	6.55 ${}^{b}$	0.29 ${}^{b}$	51.44 ${}^{a}$
SEM	0.017	8.440	3.350	0.640	0.027	2.050
P -value	0.001	0.001	0.004	0.001	0.016	0.017

${}^{\text{a, b, c}}$ The column average is significantly different. SEM: standard error of the means.

Saki et al. (2014) found that the addition of 5 % pomegranate seed meal to laying-hen rations had no effect on total serum antioxidants but increased the MDA value. The researchers have suggested that pomegranate seed oil contains a high amount of polyunsaturated fatty acids (PUFAs), long-chain fatty acids that are more sensitive to oxidation, and that the plasma MDA level may therefore be increased. In a study on rats, it was reported that 50 $\mathrm{mg}\;{\mathrm{kg}}^{-1}$ of pomegranate peel extract added to the ration significantly protected catalase, peroxidase and SOD enzyme activities (Chidambara Murthy et al., 2002). Similarly, Saha and Ghosh (2009) reported that punicic acid increased the activity of catalase, glutathione and SOD in rats.

In this study, it was determined that the TAS value was quite low in the 1 $\mathrm{mL}\;{\mathrm{kg}}^{-1}$ PSO group. Rojo-Gutiérrez et al. (2021) reported that pomegranate seed oil showed 91.29 % scavenging activity against DPPH radical. The reason for the decrease in TAS value can be explained by the antioxidant property of PSO.

The addition of PSO to the diet had no effect on myristic, palmitic, oleic, linoleic, gamma linolenic, alpha linolenic, arachidonic, docosahexaenoic acid (DHA), and monounsaturated fatty acids (MUFA) (Table 7). It was observed that the addition of PSO decreased the palmitioleic acid ratio and increased the stearic acid, CLA, punicic acid, eicosapentaenoic acid (EPA), SFA, and PUFA. The highest stearic acid and SFA values were found in the 1 $\mathrm{mL}\;{\mathrm{kg}}^{-1}$ PSO group, while the highest CLA, CLnA (punicic acid), and PUFA values were found in the 1.5 $\mathrm{mL}\;{\mathrm{kg}}^{-1}$ PSO group.

**Table 7:** Effects of PSO on the fatty acid composition in the yolk of laying hens.

Fatty acids	Control	PSO	PSO	PSO	SEM	P value
		0.5 $\mathrm{mL}\;{\mathrm{kg}}^{-1}$	1 $\mathrm{mL}\;{\mathrm{kg}}^{-1}$	1.5 $\mathrm{mL}\;{\mathrm{kg}}^{-1}$		
Miristic (C14 : 0)	1.51	1.15	1.39	1.19	1.305	0.072
Palmitic (C16 : 0)	28.35	28.16	27.33	27.30	0.416	0.822
Palmitioleic (C16 : 1c9)	4.96 ${}^{a}$	4.47 ${}^{\text{ab}}$	3.69 ${}^{c}$	4.58 ${}^{\text{ab}}$	0.183	0.001
Stearic (C18 : 0)	10.41 ${}^{c}$	13.93 ${}^{b}$	17.23 ${}^{a}$	15.16 ${}^{\text{ab}}$	0.976	0.012
Oleic (C18 : 1c9)	34.15	31.56	31.19	29.22	0.840	0.231
Linoleic (C18 : 2c9,c12)	11.57	11.98	9.56	13.07	0.520	0.053
Y-linolenic (C18 : 3c6,c9,c12)	0.24	0.23	0.25	0.26	0.011	0.873
Alfalinolenic (C18 : 3c9,c12,c15)	0.33	0.31	0.26	0.41	0.022	0.089
CLA (C18 : 2c9,t11/t10,c12)	0.010 ${}^{c}$	0.49 ${}^{b}$	0.52 ${}^{b}$	0.63 ${}^{a}$	0.020	0.005
Eicosatrienoic (C20 : 3c8,c11,c14)	0.24 ${}^{b}$	0.26 ${}^{b}$	0.33 ${}^{a}$	0.29 ${}^{\text{ab}}$	0.013	0.012
Arachidonic (C20 : 4c5,c8,c11,c14)	2.44	2.33	3.05	2.15	0.140	0.097
CLnA (76 % Punicic acid C18 : 3c9,t11,c13)	0 ${}^{d}$	0.59 ${}^{c}$	1.28 ${}^{b}$	1.98 ${}^{a}$	0.070	0.001
EPA (C20 : c5,c8,c11,c14,c17)	0.79 ${}^{c}$	0.94 ${}^{b}$	1.02 ${}^{a}$	0.88 ${}^{b}$	0.060	0.043
DHA (C22 : 6c4,c7,c10,c13,c16,c19)	0.93	0.90	0.98	0.89	0.020	0.482
SFA	40.09 ${}^{b}$	43.91 ${}^{a}$	46.21 ${}^{a}$	42.86 ${}^{\text{ab}}$	0.800	0.023
MUFA	37.79	35.15	34.80	36.68	0.740	0.521
PUFA	15.79 ${}^{c}$	18.03 ${}^{b}$	17.25 ${}^{b}$	20.56 ${}^{a}$	0.550	0.000

${}^{\text{a, b, c, d}}$ The raw average is significantly different. SEM: standard error of the means.

In this study, it was observed that the addition of PSO to the ration increased EPA and the CLA ratio, and an increase in the SEFA ratio was observed in parallel with the increase in the CLA ratio. Ngo Njembe et al. (2021b) reported that the addition of PSO to the laying-hen diets decreased the MUFA ratio statistically significantly, while the SFA ratio was numerically higher than the control group. Trans 10 cis 12 CLA isomer decreases MUFA and increases SFA and sterol coenzyme A desaturase enzyme activity in the liver. This enzyme catalyzes the double bond between the c9 and c10 atoms of palmitioleic and oleic fatty acids, which convert to 16 : 0 and 18 : 0 (Smith et al., 2002). Similar to this study, Kostogrys et al. (2017) reported that the ratio of SFA and CLA increased and MUFA ratio decreased with the increase of punicic acid level in laying-hen diets. Stearoyl-CoA desaturase (SCD) is an enzyme that catalyzes the Δ 9-cis desaturation of fatty acyl-CoA substrates and converts palmitoyl- and stearoyl-CoA to palmitoleoyl- and oleoyl-CoA, respectively. Several studies have shown that CLnA inhibits Δ 9 desaturation in the liver through inhibition of stearoyl-CoA desaturase activity and reduction of SCD-1 mRNA expression (Arao et al., 2004; Shinohara et al., 2012a). In the present study, it was determined that the CLnA level increased with the increase PSO level in the diet, and it is thought that increasing CLnA in the diet containing high PSO inhibits the SCD enzyme, and accordingly, MUFA decreases and SFA increases. Many researchers have suggested that the fatty acid profile of egg yolks is affected by dietary fatty acid composition (Bölükbaşı et al., 2006; Kostogrys et al., 2017).

Pomegranate seed oil is very rich in polyunsaturated fatty acid, about 55 %–78 % of which is punicic acid (cis 9, trans 11, cis 13 acid). The CLnA (containing 76 % punicic acid) ratio in egg yolk increased with the increase of the PSO ratio in the diet in this study. Similarly, it has been reported that the addition of different levels of punicic acid (Kostogrys et al., 2017) to the diet increases the rate of punicic acid in egg yolks. Ngo Njembe et al. (2021a) reported that the addition of PSO to laying-hen rations increased the ratio of CLA and punicic acid. The authors speculated that the inclusion of PSO in the laying-hen diet also allowed some degree of accumulation of another isomer of CLA (t9,t11-CLA), possibly derived from the metabolism of CLnA, apart from the punicic acid found in PSO. Ngo Njembe et al. (2021a) show that laying hens can efficiently transform various CLnA isomers with a D13 double bond. In several previous studies, it has been reported that CLnA isomers (punicic acid, α -eleostearic acid) can be converted to rumenic acid (t9, t11-CLA) via a Δ 13 reductase in animal and human tissues (Suzuki et al., 2006; Schneider et al., 2013).

It has been reported that egg quality decreased in some studies carried out to increase CLA and CLnA levels in eggs by adding chicken meal, fish meal, fish oil, and flaxseed to rations (Hayat et al., 2010; Dunn-Horrocks et al., 2011). However, in a limited number of studies on PSO, it has been reported that the amount of CLA and CLnA in the egg yolk will be increased without reducing egg quality (Kostogrys et al., 2017).

## 4 Conclusion

In conclusion, it was observed that the addition of PSO to rations decreased the feed consumption, especially in the 1 $\mathrm{mL}\;{\mathrm{kg}}^{-1}$ PSO group, and it increased the egg weight in this study. It was determined that the shelf life of eggs was positively affected in all groups to which PSO was added. As a result, it was found that the shelf life was positively affected and the levels of CLnA (punicic acid) and CLA increased in eggs without reducing egg quality with the addition of PSO.

## Data Availability

The data presented in this study are available free of charge for any user upon reasonable request from the corresponding author.
